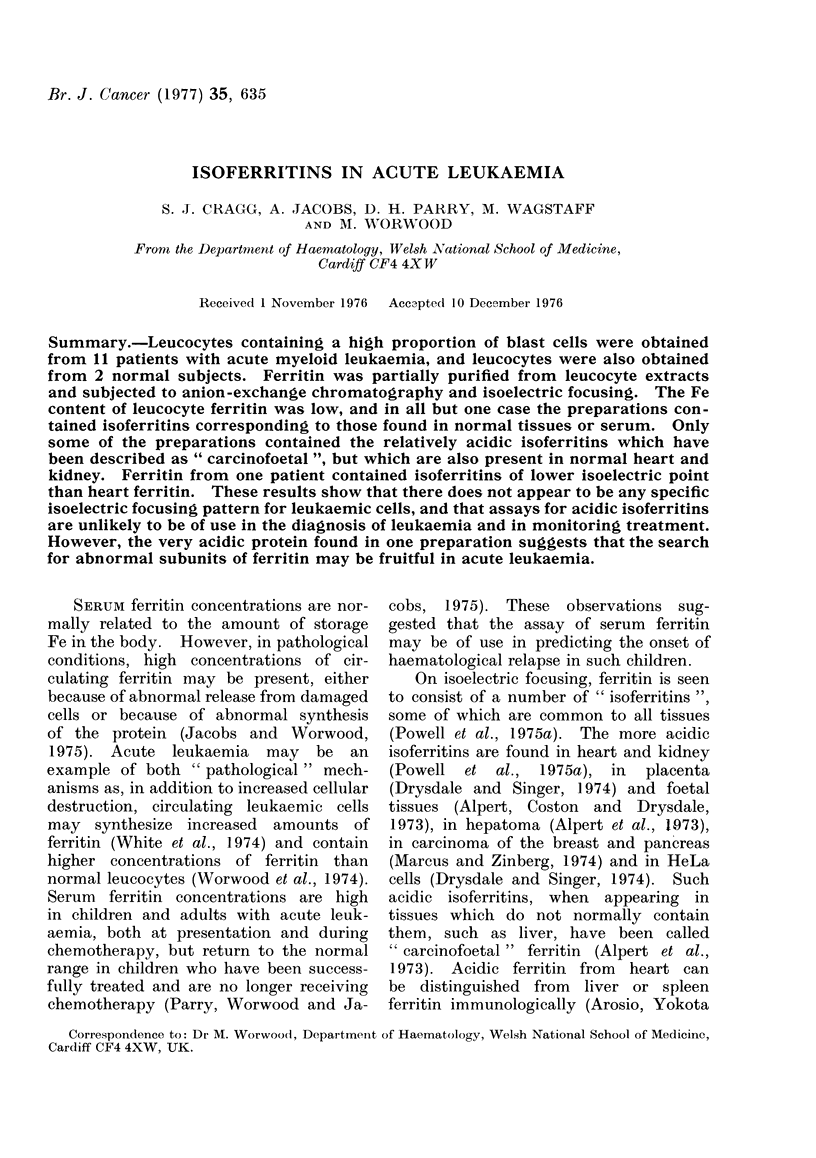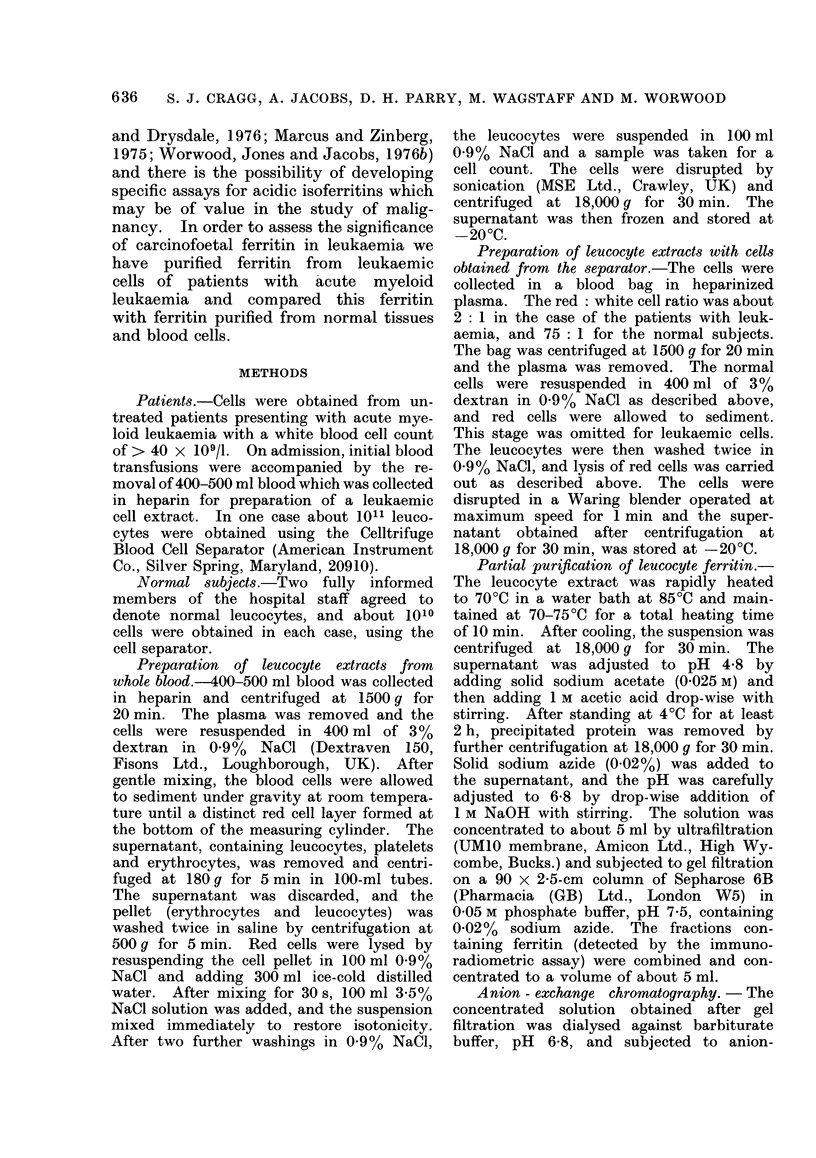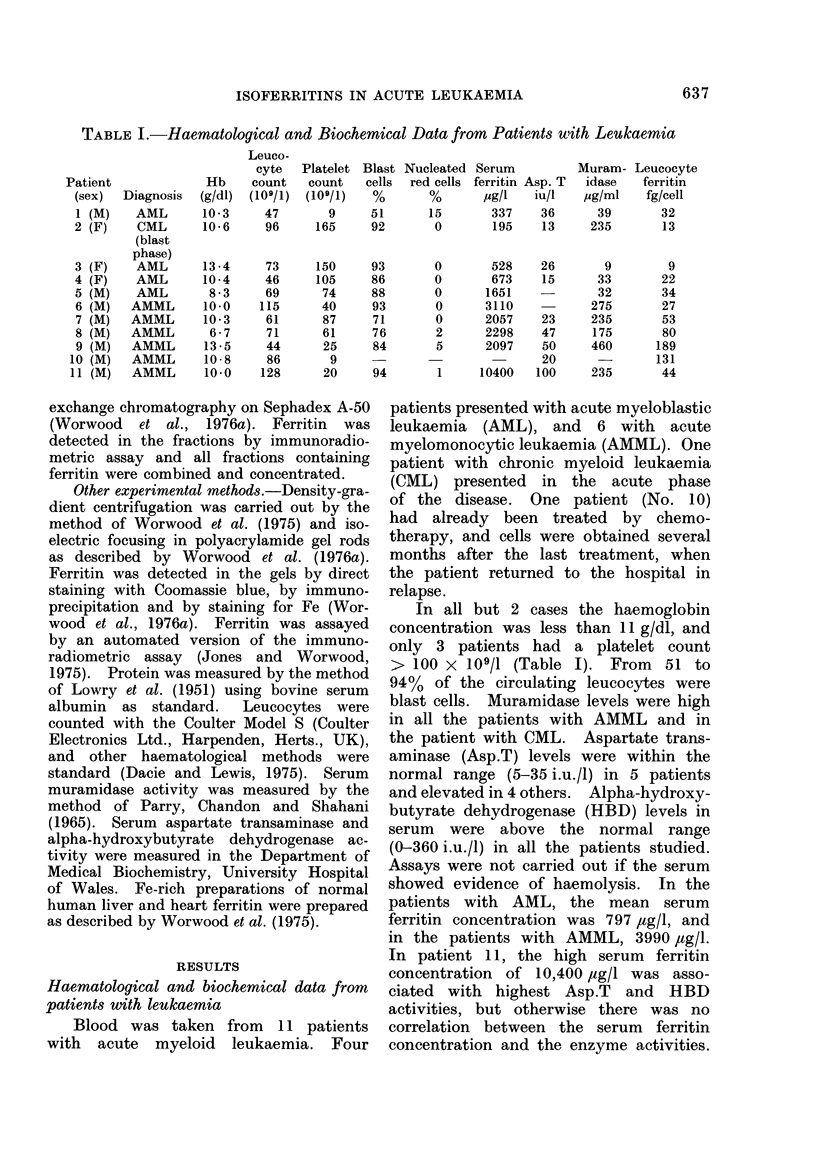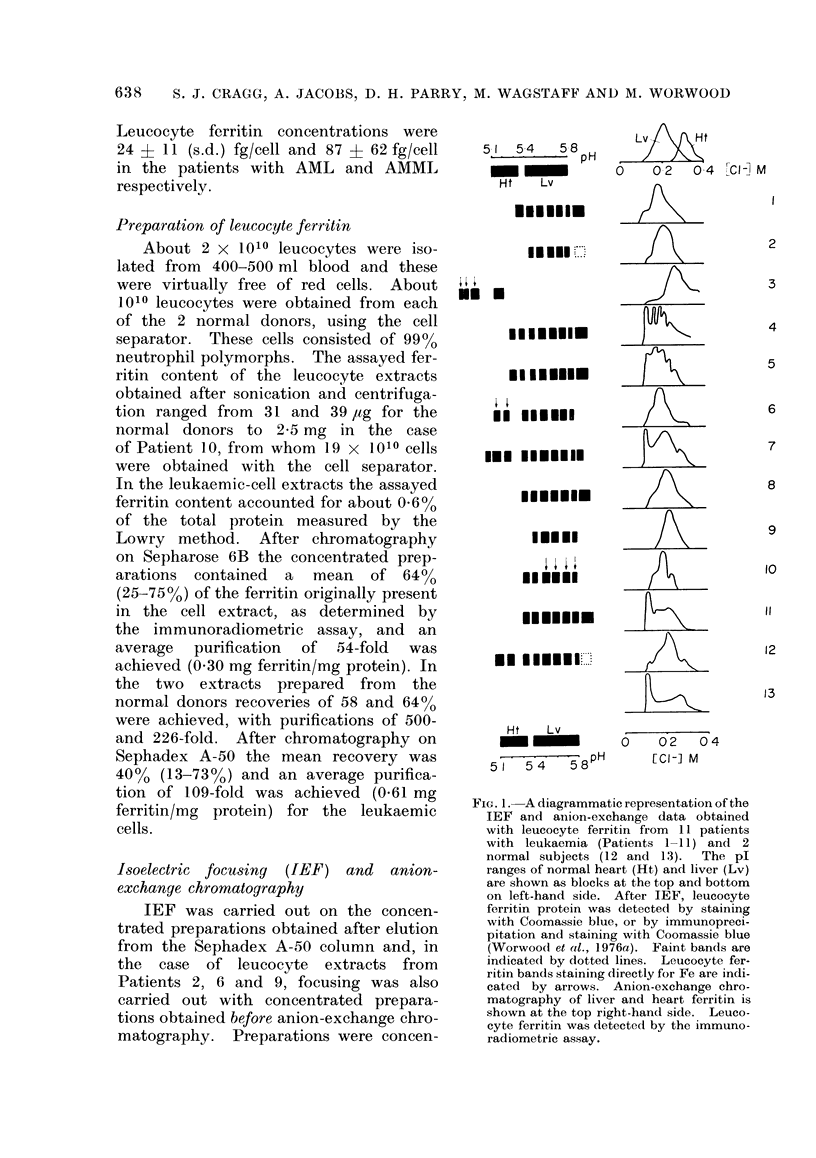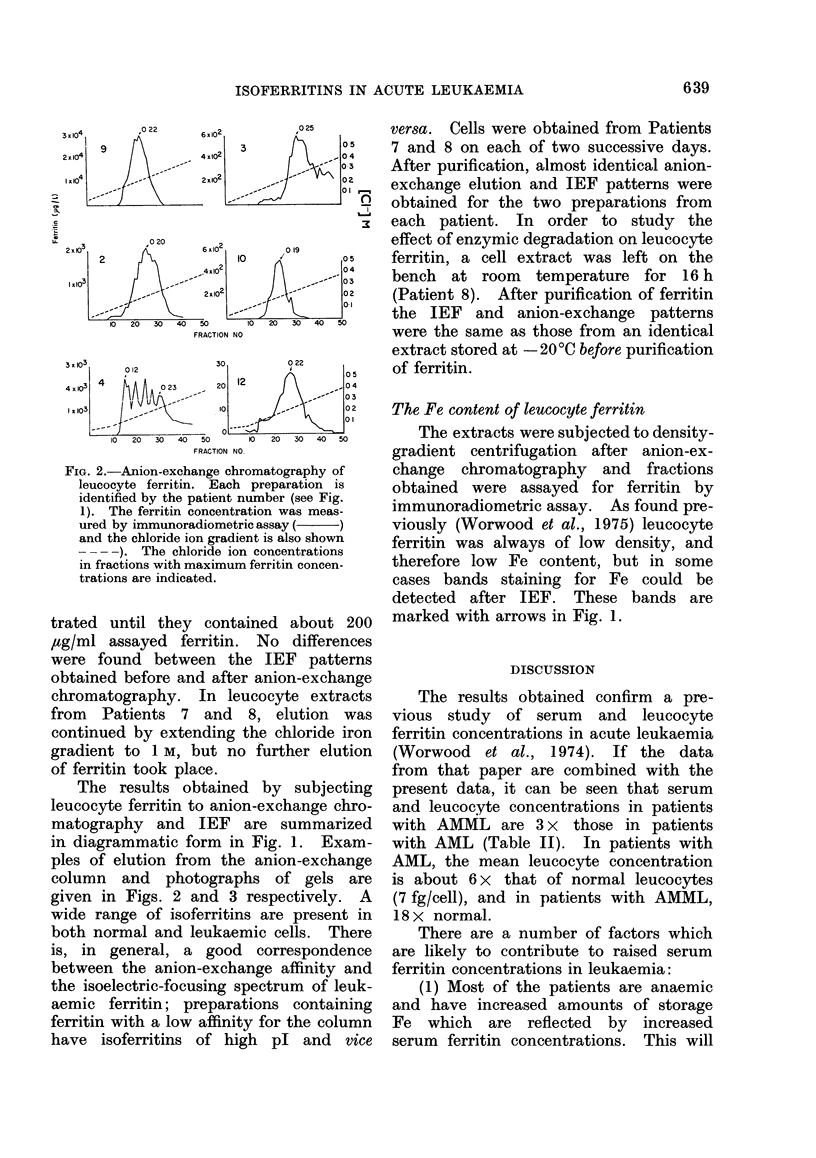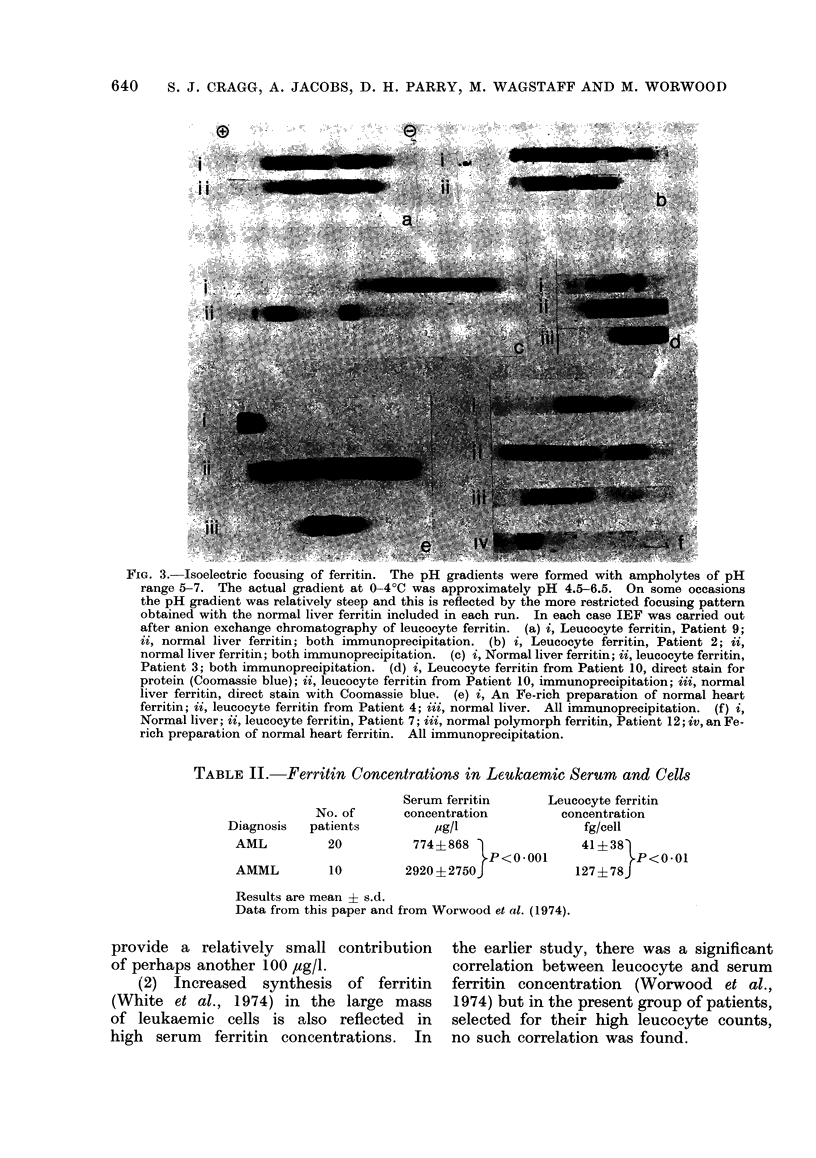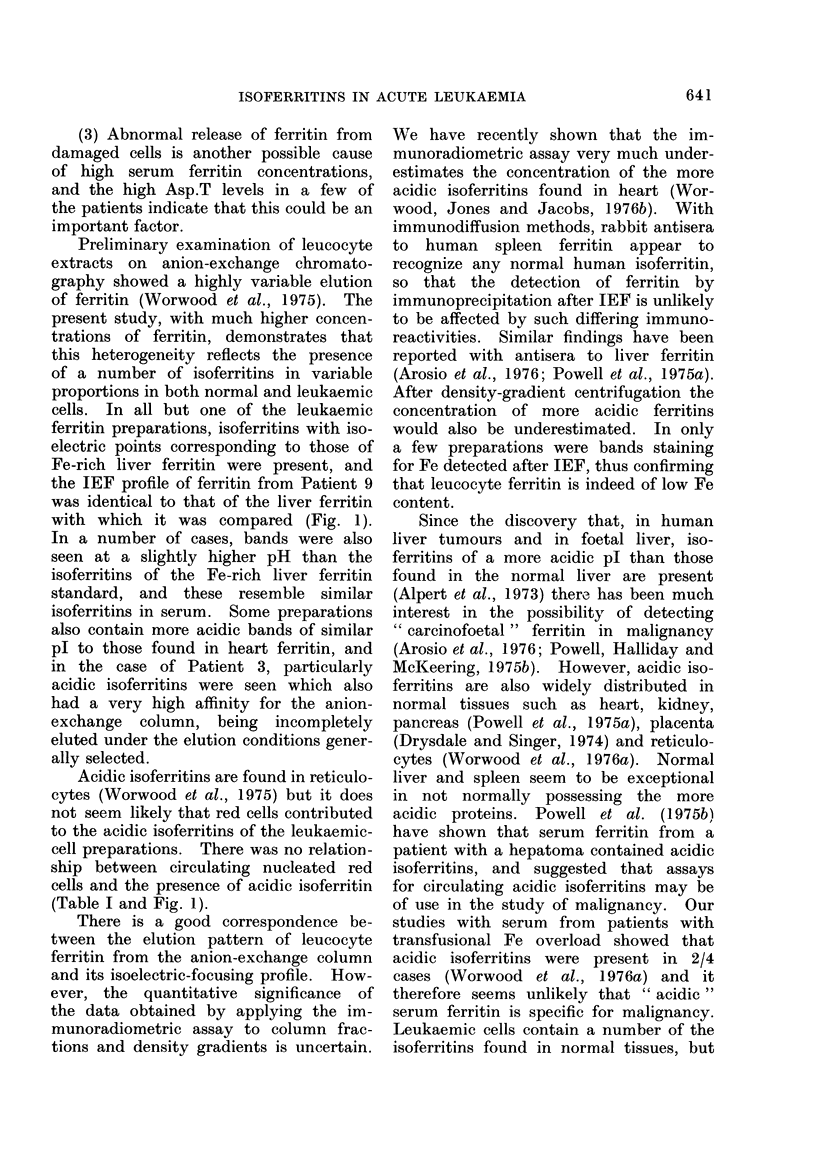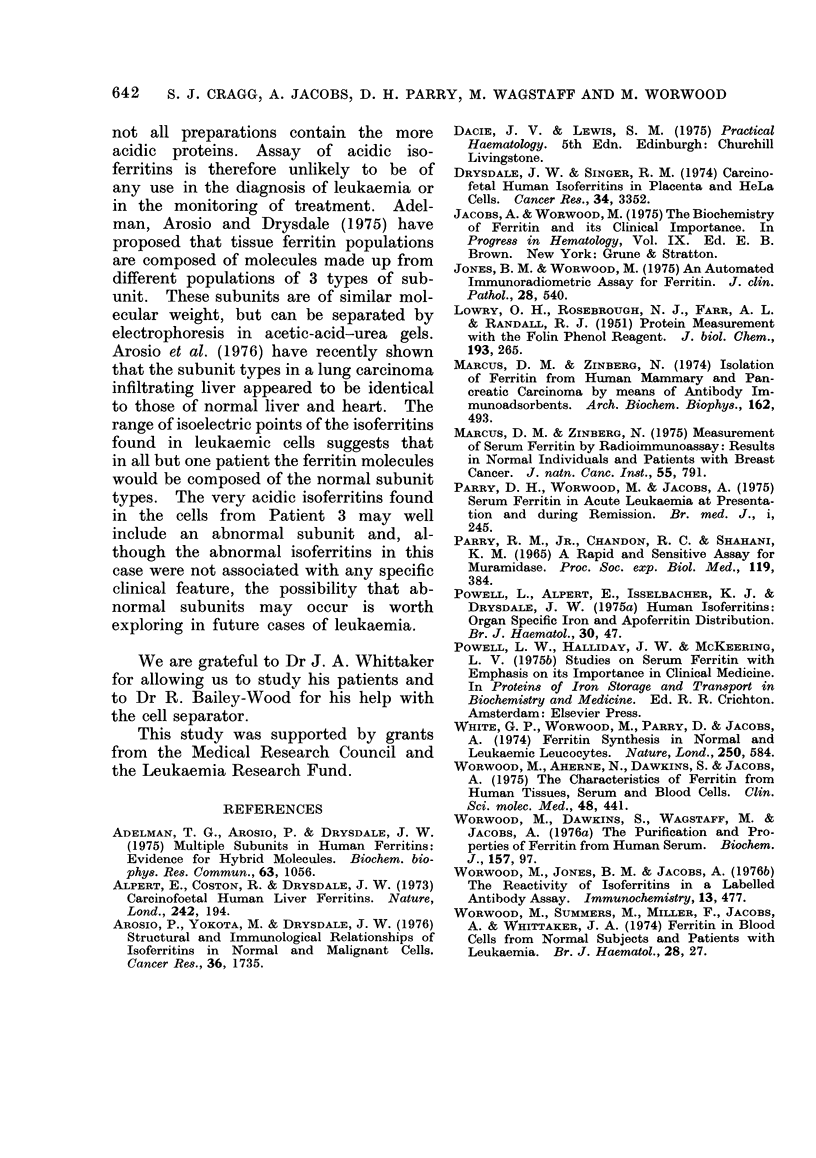# Isoferritins in acute leukaemia.

**DOI:** 10.1038/bjc.1977.99

**Published:** 1977-05

**Authors:** S. J. Cragg, A. Jacobs, D. H. Parry, M. Wagstaff, M. Worwood

## Abstract

**Images:**


Br. J. Cancer (1977) 35, 635

### ISOFERRITINS IN ACUTE LEUKAEMIA

S. J. CRAGG, A. JACOBS, D. H. PARRY, M. WAGSTAFF AND M. WORWOOD

*From the Department of Haematology, Welsh National School of Medicine, Cardiff CF4 4XW*

Received 1 November 1976 Accepted 10 December 1976

**Summary.—Leucocytes containing a high proportion of blast cells were obtained from 11 patients with acute myeloid leukaemia, and leucocytes were also obtained from 2 normal subjects. Ferritin was partially purified from leucocyte extracts and subjected to anion-exchange chromatography and isoelectric focusing. The Fe content of leucocyte ferritin was low, and in all but one case the preparations contained isoferritins corresponding to those found in normal tissues or serum. Only some of the preparations contained the relatively acidic isoferritins which have been described as " carcinofoetal ", but which are also present in normal heart and kidney. Ferritin from one patient contained isoferritins of lower isoelectric point than heart ferritin. These results show that there does not appear to be any specific isoelectric focusing pattern for leukaemic cells, and that assays for acidic isoferritins are unlikely to be of use in the diagnosis of leukaemia and in monitoring treatment. However, the very acidic protein found in one preparation suggests that the search for abnormal subunits of ferritin may be fruitful in acute leukaemia.**

SERUM ferritin concentrations are normally related to the amount of storage Fe in the body. However, in pathological conditions, high concentrations of circulating ferritin may be present, either because of abnormal release from damaged cells or because of abnormal synthesis of the protein (Jacobs and Worwood, 1975). Acute leukaemia may be an example of both " pathological " mechanisms as, in addition to increased cellular destruction, circulating leukaemic cells may synthesize increased amounts of ferritin (White *et al.*, 1974) and contain higher concentrations of ferritin than normal leucocytes (Worwood *et al.*, 1974). Serum ferritin concentrations are high in children and adults with acute leukaemia, both at presentation and during chemotherapy, but return to the normal range in children who have been successfully treated and are no longer receiving chemotherapy (Parry, Worwood and Jacobs, 1975). These observations suggested that the assay of serum ferritin may be of use in predicting the onset of haematological relapse in such children.

On isoelectric focusing, ferritin is seen to consist of a number of " isoferritins ", some of which are common to all tissues (Powell *et al.*, 1975*a*). The more acidic isoferritins are found in heart and kidney (Powell *et al.*, 1975*a*), in placenta (Drysdale and Singer, 1974) and foetal tissues (Alpert, Coston and Drysdale, 1973), in hepatoma (Alpert *et al.*, 1973), in carcinoma of the breast and pancreas (Marcus and Zinberg, 1974) and in HeLa cells (Drysdale and Singer, 1974). Such acidic isoferritins, when appearing in tissues which do not normally contain them, such as liver, have been called " carcinofoetal " ferritin (Alpert *et al.*, 1973). Acidic ferritin from heart can be distinguished from liver or spleen ferritin immunologically (Arosio, Yokota

Correspondence to: Dr M. Worwood, Department of Haematology, Welsh National School of Medicine, Cardiff CF4 4XW, UK.

and Drysdale, 1976; Marcus and Zinberg, 1975; Worwood, Jones and Jacobs, 1976*b*) and there is the possibility of developing specific assays for acidic isoferritins which may be of value in the study of malignancy. In order to assess the significance of carcinofoetal ferritin in leukaemia we have purified ferritin from leukaemic cells of patients with acute myeloid leukaemia and compared this ferritin with ferritin purified from normal tissues and blood cells.

### METHODS

*Patients.*—Cells were obtained from untreated patients presenting with acute myeloid leukaemia with a white blood cell count of $> 40 \times 10^9$/l. On admission, initial blood transfusions were accompanied by the removal of 400–500 ml blood which was collected in heparin for preparation of a leukaemic cell extract. In one case about $10^{11}$ leucocytes were obtained using the Celltrifuge Blood Cell Separator (American Instrument Co., Silver Spring, Maryland, 20910).

*Normal subjects.*—Two fully informed members of the hospital staff agreed to denote normal leucocytes, and about $10^{10}$ cells were obtained in each case, using the cell separator.

*Preparation of leucocyte extracts from whole blood.*—400–500 ml blood was collected in heparin and centrifuged at 1500 *g* for 20 min. The plasma was removed and the cells were resuspended in 400 ml of 3% dextran in 0·9% NaCl (Dextraven 150, Fisons Ltd., Loughborough, UK). After gentle mixing, the blood cells were allowed to sediment under gravity at room temperature until a distinct red cell layer formed at the bottom of the measuring cylinder. The supernatant, containing leucocytes, platelets and erythrocytes, was removed and centrifuged at 180 *g* for 5 min in 100-ml tubes. The supernatant was discarded, and the pellet (erythrocytes and leucocytes) was washed twice in saline by centrifugation at 500 *g* for 5 min. Red cells were lysed by resuspending the cell pellet in 100 ml 0·9% NaCl and adding 300 ml ice-cold distilled water. After mixing for 30 s, 100 ml 3·5% NaCl solution was added, and the suspension mixed immediately to restore isotonicity. After two further washings in 0·9% NaCl, the leucocytes were suspended in 100 ml 0·9% NaCl and a sample was taken for a cell count. The cells were disrupted by sonication (MSE Ltd., Crawley, UK) and centrifuged at 18,000 *g* for 30 min. The supernatant was then frozen and stored at −20°C.

*Preparation of leucocyte extracts with cells obtained from the separator.*—The cells were collected in a blood bag in heparinized plasma. The red : white cell ratio was about 2 : 1 in the case of the patients with leukaemia, and 75 : 1 for the normal subjects. The bag was centrifuged at 1500 *g* for 20 min and the plasma was removed. The normal cells were resuspended in 400 ml of 3% dextran in 0·9% NaCl as described above, and red cells were allowed to sediment. This stage was omitted for leukaemic cells. The leucocytes were then washed twice in 0·9% NaCl, and lysis of red cells was carried out as described above. The cells were disrupted in a Waring blender operated at maximum speed for 1 min and the supernatant obtained after centrifugation at 18,000 *g* for 30 min, was stored at −20°C.

*Partial purification of leucocyte ferritin.*—The leucocyte extract was rapidly heated to 70°C in a water bath at 85°C and maintained at 70–75°C for a total heating time of 10 min. After cooling, the suspension was centrifuged at 18,000 *g* for 30 min. The supernatant was adjusted to pH 4·8 by adding solid sodium acetate (0·025 M) and then adding 1 M acetic acid drop-wise with stirring. After standing at 4°C for at least 2 h, precipitated protein was removed by further centrifugation at 18,000 *g* for 30 min. Solid sodium azide (0·02%) was added to the supernatant, and the pH was carefully adjusted to 6·8 by drop-wise addition of 1 M NaOH with stirring. The solution was concentrated to about 5 ml by ultrafiltration (UM10 membrane, Amicon Ltd., High Wycombe, Bucks.) and subjected to gel filtration on a 90 × 2·5-cm column of Sepharose 6B (Pharmacia (GB) Ltd., London W5) in 0·05 M phosphate buffer, pH 7·5, containing 0·02% sodium azide. The fractions containing ferritin (detected by the immunoradiometric assay) were combined and concentrated to a volume of about 5 ml.

*Anion - exchange chromatography.* — The concentrated solution obtained after gel filtration was dialysed against barbiturate buffer, pH 6·8, and subjected to anion-

TABLE I.—*Haematological and Biochemical Data from Patients with Leukaemia*

| Patient (sex) | Diagnosis | Hb (g/dl) | Leucocyte count ($10^9$/l) | Platelet count ($10^9$/l) | Blast cells % | Nucleated red cells % | Serum ferritin μg/l | Asp. T iu/l | Muramidase μg/ml | Leucocyte ferritin fg/cell |
|---|---|---|---|---|---|---|---|---|---|---|
| 1 (M) | AML | 10·3 | 47 | 9 | 51 | 15 | 337 | 36 | 39 | 32 |
| 2 (F) | CML (blast phase) | 10·6 | 96 | 165 | 92 | 0 | 195 | 13 | 235 | 13 |
| 3 (F) | AML | 13·4 | 73 | 150 | 93 | 0 | 528 | 26 | 9 | 9 |
| 4 (F) | AML | 10·4 | 46 | 105 | 86 | 0 | 673 | 15 | 33 | 22 |
| 5 (M) | AML | 8·3 | 69 | 74 | 88 | 0 | 1651 | — | 32 | 34 |
| 6 (M) | AMML | 10·0 | 115 | 40 | 93 | 0 | 3110 | — | 275 | 27 |
| 7 (M) | AMML | 10·3 | 61 | 87 | 71 | 0 | 2057 | 23 | 235 | 53 |
| 8 (M) | AMML | 6·7 | 71 | 61 | 76 | 2 | 2298 | 47 | 175 | 80 |
| 9 (M) | AMML | 13·5 | 44 | 25 | 84 | 5 | 2097 | 50 | 460 | 189 |
| 10 (M) | AMML | 10·8 | 86 | 9 | — | — | — | 20 | — | 131 |
| 11 (M) | AMML | 10·0 | 128 | 20 | 94 | 1 | 10400 | 100 | 235 | 44 |

exchange chromatography on Sephadex A-50 (Worwood *et al.*, 1976*a*). Ferritin was detected in the fractions by immunoradiometric assay and all fractions containing ferritin were combined and concentrated.

*Other experimental methods.*—Density-gradient centrifugation was carried out by the method of Worwood *et al.* (1975) and isoelectric focusing in polyacrylamide gel rods as described by Worwood *et al.* (1976*a*). Ferritin was detected in the gels by direct staining with Coomassie blue, by immunoprecipitation and by staining for Fe (Worwood *et al.*, 1976*a*). Ferritin was assayed by an automated version of the immunoradiometric assay (Jones and Worwood, 1975). Protein was measured by the method of Lowry *et al.* (1951) using bovine serum albumin as standard. Leucocytes were counted with the Coulter Model S (Coulter Electronics Ltd., Harpenden, Herts., UK), and other haematological methods were standard (Dacie and Lewis, 1975). Serum muramidase activity was measured by the method of Parry, Chandon and Shahani (1965). Serum aspartate transaminase and alpha-hydroxybutyrate dehydrogenase activity were measured in the Department of Medical Biochemistry, University Hospital of Wales. Fe-rich preparations of normal human liver and heart ferritin were prepared as described by Worwood *et al.* (1975).

### RESULTS

### *Haematological and biochemical data from patients with leukaemia*

Blood was taken from 11 patients with acute myeloid leukaemia. Four patients presented with acute myeloblastic leukaemia (AML), and 6 with acute myelomonocytic leukaemia (AMML). One patient with chronic myeloid leukaemia (CML) presented in the acute phase of the disease. One patient (No. 10) had already been treated by chemotherapy, and cells were obtained several months after the last treatment, when the patient returned to the hospital in relapse.

In all but 2 cases the haemoglobin concentration was less than 11 g/dl, and only 3 patients had a platelet count $> 100 \times 10^9$/l (Table I). From 51 to 94% of the circulating leucocytes were blast cells. Muramidase levels were high in all the patients with AMML and in the patient with CML. Aspartate transaminase (Asp.T) levels were within the normal range (5–35 i.u./l) in 5 patients and elevated in 4 others. Alpha-hydroxybutyrate dehydrogenase (HBD) levels in serum were above the normal range (0–360 i.u./l) in all the patients studied. Assays were not carried out if the serum showed evidence of haemolysis. In the patients with AML, the mean serum ferritin concentration was 797 μg/l, and in the patients with AMML, 3990 μg/l. In patient 11, the high serum ferritin concentration of 10,400 μg/l was associated with highest Asp.T and HBD activities, but otherwise there was no correlation between the serum ferritin concentration and the enzyme activities.

Leucocyte ferritin concentrations were 24 ± 11 (s.d.) fg/cell and 87 ± 62 fg/cell in the patients with AML and AMML respectively.

*Preparation of leucocyte ferritin*

About $2 \times 10^{10}$ leucocytes were isolated from 400–500 ml blood and these were virtually free of red cells. About $10^{10}$ leucocytes were obtained from each of the 2 normal donors, using the cell separator. These cells consisted of 99% neutrophil polymorphs. The assayed ferritin content of the leucocyte extracts obtained after sonication and centrifugation ranged from 31 and 39 μg for the normal donors to 2·5 mg in the case of Patient 10, from whom $19 \times 10^{10}$ cells were obtained with the cell separator. In the leukaemic-cell extracts the assayed ferritin content accounted for about 0·6% of the total protein measured by the Lowry method. After chromatography on Sepharose 6B the concentrated preparations contained a mean of 64% (25–75%) of the ferritin originally present in the cell extract, as determined by the immunoradiometric assay, and an average purification of 54-fold was achieved (0·30 mg ferritin/mg protein). In the two extracts prepared from the normal donors recoveries of 58 and 64% were achieved, with purifications of 500- and 226-fold. After chromatography on Sephadex A-50 the mean recovery was 40% (13–73%) and an average purification of 109-fold was achieved (0·61 mg ferritin/mg protein) for the leukaemic cells.

*Isoelectric focusing (IEF) and anion-exchange chromatography*

IEF was carried out on the concentrated preparations obtained after elution from the Sephadex A-50 column and, in the case of leucocyte extracts from Patients 2, 6 and 9, focusing was also carried out with concentrated preparations obtained *before* anion-exchange chromatography. Preparations were concen-

FIG. 1.—A diagrammatic representation of the IEF and anion-exchange data obtained with leucocyte ferritin from 11 patients with leukaemia (Patients 1–11) and 2 normal subjects (12 and 13). The pI ranges of normal heart (Ht) and liver (Lv) are shown as blocks at the top and bottom on left-hand side. After IEF, leucocyte ferritin protein was detected by staining with Coomassie blue, or by immunoprecipitation and staining with Coomassie blue (Worwood *et al.*, 1976*a*). Faint bands are indicated by dotted lines. Leucocyte ferritin bands staining directly for Fe are indicated by arrows. Anion-exchange chromatography of liver and heart ferritin is shown at the top right-hand side. Leucocyte ferritin was detected by the immunoradiometric assay.

FIG. 2.—Anion-exchange chromatography of leucocyte ferritin. Each preparation is identified by the patient number (see Fig. 1). The ferritin concentration was measured by immunoradiometric assay (———) and the chloride ion gradient is also shown ( – – – –). The chloride ion concentrations in fractions with maximum ferritin concentrations are indicated.

trated until they contained about 200 μg/ml assayed ferritin. No differences were found between the IEF patterns obtained before and after anion-exchange chromatography. In leucocyte extracts from Patients 7 and 8, elution was continued by extending the chloride iron gradient to 1 M, but no further elution of ferritin took place.

The results obtained by subjecting leucocyte ferritin to anion-exchange chromatography and IEF are summarized in diagrammatic form in Fig. 1. Examples of elution from the anion-exchange column and photographs of gels are given in Figs. 2 and 3 respectively. A wide range of isoferritins are present in both normal and leukaemic cells. There is, in general, a good correspondence between the anion-exchange affinity and the isoelectric-focusing spectrum of leukaemic ferritin; preparations containing ferritin with a low affinity for the column have isoferritins of high pI and *vice versa*. Cells were obtained from Patients 7 and 8 on each of two successive days. After purification, almost identical anion-exchange elution and IEF patterns were obtained for the two preparations from each patient. In order to study the effect of enzymic degradation on leucocyte ferritin, a cell extract was left on the bench at room temperature for 16 h (Patient 8). After purification of ferritin the IEF and anion-exchange patterns were the same as those from an identical extract stored at −20 °C *before* purification of ferritin.

### *The Fe content of leucocyte ferritin*

The extracts were subjected to density-gradient centrifugation after anion-exchange chromatography and fractions obtained were assayed for ferritin by immunoradiometric assay. As found previously (Worwood *et al.*, 1975) leucocyte ferritin was always of low density, and therefore low Fe content, but in some cases bands staining for Fe could be detected after IEF. These bands are marked with arrows in Fig. 1.

### DISCUSSION

The results obtained confirm a previous study of serum and leucocyte ferritin concentrations in acute leukaemia (Worwood *et al.*, 1974). If the data from that paper are combined with the present data, it can be seen that serum and leucocyte concentrations in patients with AMML are 3× those in patients with AML (Table II). In patients with AML, the mean leucocyte concentration is about 6× that of normal leucocytes (7 fg/cell), and in patients with AMML, 18× normal.

There are a number of factors which are likely to contribute to raised serum ferritin concentrations in leukaemia:

(1) Most of the patients are anaemic and have increased amounts of storage Fe which are reflected by increased serum ferritin concentrations. This will

FIG. 3.—Isoelectric focusing of ferritin. The pH gradients were formed with ampholytes of pH range 5–7. The actual gradient at 0–4°C was approximately pH 4.5–6.5. On some occasions the pH gradient was relatively steep and this is reflected by the more restricted focusing pattern obtained with the normal liver ferritin included in each run. In each case IEF was carried out after anion exchange chromatography of leucocyte ferritin. (a) *i*, Leucocyte ferritin, Patient 9; *ii*, normal liver ferritin; both immunoprecipitation. (b) *i*, Leucocyte ferritin, Patient 2; *ii*, normal liver ferritin; both immunoprecipitation. (c) *i*, Normal liver ferritin; *ii*, leucocyte ferritin, Patient 3; both immunoprecipitation. (d) *i*, Leucocyte ferritin from Patient 10, direct stain for protein (Coomassie blue); *ii*, leucocyte ferritin from Patient 10, immunoprecipitation; *iii*, normal liver ferritin, direct stain with Coomassie blue. (e) *i*, An Fe-rich preparation of normal heart ferritin; *ii*, leucocyte ferritin from Patient 4; *iii*, normal liver. All immunoprecipitation. (f) *i*, Normal liver; *ii*, leucocyte ferritin, Patient 7; *iii*, normal polymorph ferritin, Patient 12; *iv*, an Fe-rich preparation of normal heart ferritin. All immunoprecipitation.

TABLE II.—*Ferritin Concentrations in Leukaemic Serum and Cells*

| Diagnosis | No. of patients | Serum ferritin concentration μg/l | | Leucocyte ferritin concentration fg/cell | |
|---|---|---|---|---|---|
| AML | 20 | 774±868 | $P<0\cdot001$ | 41±38 | $P<0\cdot01$ |
| AMML | 10 | 2920±2750 | | 127±78 | |

Results are mean ± s.d.
Data from this paper and from Worwood *et al.* (1974).

provide a relatively small contribution of perhaps another 100 μg/l.

(2) Increased synthesis of ferritin (White *et al.*, 1974) in the large mass of leukaemic cells is also reflected in high serum ferritin concentrations. In the earlier study, there was a significant correlation between leucocyte and serum ferritin concentration (Worwood *et al.*, 1974) but in the present group of patients, selected for their high leucocyte counts, no such correlation was found.

(3) Abnormal release of ferritin from damaged cells is another possible cause of high serum ferritin concentrations, and the high Asp.T levels in a few of the patients indicate that this could be an important factor.

Preliminary examination of leucocyte extracts on anion-exchange chromatography showed a highly variable elution of ferritin (Worwood *et al.*, 1975). The present study, with much higher concentrations of ferritin, demonstrates that this heterogeneity reflects the presence of a number of isoferritins in variable proportions in both normal and leukaemic cells. In all but one of the leukaemic ferritin preparations, isoferritins with isoelectric points corresponding to those of Fe-rich liver ferritin were present, and the IEF profile of ferritin from Patient 9 was identical to that of the liver ferritin with which it was compared (Fig. 1). In a number of cases, bands were also seen at a slightly higher pH than the isoferritins of the Fe-rich liver ferritin standard, and these resemble similar isoferritins in serum. Some preparations also contain more acidic bands of similar pI to those found in heart ferritin, and in the case of Patient 3, particularly acidic isoferritins were seen which also had a very high affinity for the anion-exchange column, being incompletely eluted under the elution conditions generally selected.

Acidic isoferritins are found in reticulocytes (Worwood *et al.*, 1975) but it does not seem likely that red cells contributed to the acidic isoferritins of the leukaemic-cell preparations. There was no relationship between circulating nucleated red cells and the presence of acidic isoferritin (Table I and Fig. 1).

There is a good correspondence between the elution pattern of leucocyte ferritin from the anion-exchange column and its isoelectric-focusing profile. However, the quantitative significance of the data obtained by applying the immunoradiometric assay to column fractions and density gradients is uncertain. We have recently shown that the immunoradiometric assay very much underestimates the concentration of the more acidic isoferritins found in heart (Worwood, Jones and Jacobs, 1976*b*). With immunodiffusion methods, rabbit antisera to human spleen ferritin appear to recognize any normal human isoferritin, so that the detection of ferritin by immunoprecipitation after IEF is unlikely to be affected by such differing immunoreactivities. Similar findings have been reported with antisera to liver ferritin (Arosio *et al.*, 1976; Powell *et al.*, 1975*a*). After density-gradient centrifugation the concentration of more acidic ferritins would also be underestimated. In only a few preparations were bands staining for Fe detected after IEF, thus confirming that leucocyte ferritin is indeed of low Fe content.

Since the discovery that, in human liver tumours and in foetal liver, isoferritins of a more acidic pI than those found in the normal liver are present (Alpert *et al.*, 1973) there has been much interest in the possibility of detecting "carcinofoetal" ferritin in malignancy (Arosio *et al.*, 1976; Powell, Halliday and McKeering, 1975*b*). However, acidic isoferritins are also widely distributed in normal tissues such as heart, kidney, pancreas (Powell *et al.*, 1975*a*), placenta (Drysdale and Singer, 1974) and reticulocytes (Worwood *et al.*, 1976*a*). Normal liver and spleen seem to be exceptional in not normally possessing the more acidic proteins. Powell *et al.* (1975*b*) have shown that serum ferritin from a patient with a hepatoma contained acidic isoferritins, and suggested that assays for circulating acidic isoferritins may be of use in the study of malignancy. Our studies with serum from patients with transfusional Fe overload showed that acidic isoferritins were present in 2/4 cases (Worwood *et al.*, 1976*a*) and it therefore seems unlikely that "acidic" serum ferritin is specific for malignancy. Leukaemic cells contain a number of the isoferritins found in normal tissues, but not all preparations contain the more acidic proteins. Assay of acidic isoferritins is therefore unlikely to be of any use in the diagnosis of leukaemia or in the monitoring of treatment. Adelman, Arosio and Drysdale (1975) have proposed that tissue ferritin populations are composed of molecules made up from different populations of 3 types of subunit. These subunits are of similar molecular weight, but can be separated by electrophoresis in acetic-acid–urea gels. Arosio *et al.* (1976) have recently shown that the subunit types in a lung carcinoma infiltrating liver appeared to be identical to those of normal liver and heart. The range of isoelectric points of the isoferritins found in leukaemic cells suggests that in all but one patient the ferritin molecules would be composed of the normal subunit types. The very acidic isoferritins found in the cells from Patient 3 may well include an abnormal subunit and, although the abnormal isoferritins in this case were not associated with any specific clinical feature, the possibility that abnormal subunits may occur is worth exploring in future cases of leukaemia.

We are grateful to Dr J. A. Whittaker for allowing us to study his patients and to Dr R. Bailey-Wood for his help with the cell separator.

This study was supported by grants from the Medical Research Council and the Leukaemia Research Fund.

### REFERENCES

Adelman, T. G., Arosio, P. & Drysdale, J. W. (1975) Multiple Subunits in Human Ferritins: Evidence for Hybrid Molecules. *Biochem. biophys. Res. Commun.*, **63**, 1056.

Alpert, E., Coston, R. & Drysdale, J. W. (1973) Carcinofoetal Human Liver Ferritins. *Nature, Lond.*, **242**, 194.

Arosio, P., Yokota, M. & Drysdale, J. W. (1976) Structural and Immunological Relationships of Isoferritins in Normal and Malignant Cells. *Cancer Res.*, **36**, 1735.

Dacie, J. V. & Lewis, S. M. (1975) *Practical Haematology.* 5th Edn. Edinburgh: Churchill Livingstone.

Drysdale, J. W. & Singer, R. M. (1974) Carcinofetal Human Isoferritins in Placenta and HeLa Cells. *Cancer Res.*, **34**, 3352.

Jacobs, A. & Worwood, M. (1975) The Biochemistry of Ferritin and its Clinical Importance. In *Progress in Hematology*, Vol. IX. Ed. E. B. Brown. New York: Grune & Stratton.

Jones, B. M. & Worwood, M. (1975) An Automated Immunoradiometric Assay for Ferritin. *J. clin. Pathol.*, **28**, 540.

Lowry, O. H., Rosebrough, N. J., Farr, A. L. & Randall, R. J. (1951) Protein Measurement with the Folin Phenol Reagent. *J. biol. Chem.*, **193**, 265.

Marcus, D. M. & Zinberg, N. (1974) Isolation of Ferritin from Human Mammary and Pancreatic Carcinoma by means of Antibody Immunoadsorbents. *Arch. Biochem. Biophys.*, **162**, 493.

Marcus, D. M. & Zinberg, N. (1975) Measurement of Serum Ferritin by Radioimmunoassay: Results in Normal Individuals and Patients with Breast Cancer. *J. natn. Canc. Inst.*, **55**, 791.

Parry, D. H., Worwood, M. & Jacobs, A. (1975) Serum Ferritin in Acute Leukaemia at Presentation and during Remission. *Br. med. J.*, i, 245.

Parry, R. M., Jr., Chandon, R. C. & Shahani, K. M. (1965) A Rapid and Sensitive Assay for Muramidase. *Proc. Soc. exp. Biol. Med.*, **119**, 384.

Powell, L., Alpert, E., Isselbacher, K. J. & Drysdale, J. W. (1975*a*) Human Isoferritins: Organ Specific Iron and Apoferritin Distribution. *Br. J. Haematol.*, **30**, 47.

Powell, L. W., Halliday, J. W. & McKeering, L. V. (1975*b*) Studies on Serum Ferritin with Emphasis on its Importance in Clinical Medicine. In *Proteins of Iron Storage and Transport in Biochemistry and Medicine.* Ed. R. R. Crichton. Amsterdam: Elsevier Press.

White, G. P., Worwood, M., Parry, D. & Jacobs, A. (1974) Ferritin Synthesis in Normal and Leukaemic Leucocytes. *Nature, Lond.*, **250**, 584.

Worwood, M., Aherne, N., Dawkins, S. & Jacobs, A. (1975) The Characteristics of Ferritin from Human Tissues, Serum and Blood Cells. *Clin. Sci. molec. Med.*, **48**, 441.

Worwood, M., Dawkins, S., Wagstaff, M. & Jacobs, A. (1976*a*) The Purification and Properties of Ferritin from Human Serum. *Biochem. J.*, **157**, 97.

Worwood, M., Jones, B. M. & Jacobs, A. (1976*b*) The Reactivity of Isoferritins in a Labelled Antibody Assay. *Immunochemistry*, **13**, 477.

Worwood, M., Summers, M., Miller, F., Jacobs, A. & Whittaker, J. A. (1974) Ferritin in Blood Cells from Normal Subjects and Patients with Leukaemia. *Br. J. Haematol.*, **28**, 27.